# The Continuum of Invasive Techniques for the Assessment of Intermediate Coronary Lesions

**DOI:** 10.3390/diagnostics12061492

**Published:** 2022-06-18

**Authors:** Nicoleta-Monica Popa-Fotea, Alexandru Scafa-Udriste, Maria Dorobantu

**Affiliations:** 1 Cardiothoracic Department, University of Medicine and Pharmacy “Carol Davila”, 8, Bulevardul Eroii Sanitari, 050474 Bucharest, Romania; alexscafa@yahoo.com (A.S.-U.); mariadorobantu@gmail.com (M.D.); 2 Emergency Clinical Hospital, 10, Calea Floreasca, 014461 Bucharest, Romania; 3 Romanian Academy, 010071 Bucharest, Romania

**Keywords:** functional assessment, intermediate lesions, fractional flow rate, intravascular ultrasound, optical coherence tomography

## Abstract

Ischemic heart disease is one of the most important causes of mortality and morbidity worldwide. Revascularization of coronary stenosis inducing ischemia, either by percutaneous or surgical intervention, significantly reduces major adverse cardiovascular events and improves quality of life. However, in cases of intermediate lesions, classified by a diameter stenosis between 50 and 90% by European guidelines and 40–70% in American counterparts with no clear evidence of ischemia, the indication of revascularization and impact is determined using various methods that altogether comprehensively evaluate the lesions. This review will discuss the various techniques to assess intermediate stenoses, highlighting indications and advantages, but also drawbacks. Fractional flow rate (FFR) and instantaneous wave-free ratio (iFR) are the gold standard for the functional evaluation of intermediate lesions, but there are clinical circumstances in which these pressure-wire-derived indices are not accurate. Complementary invasive investigations, mainly intravascular ultrasound and/or optical coherence tomography, offer parameters that can be correlated with FFR/iFR and additional insights into the morphology of the plaque guiding the eventual percutaneous intervention in terms of length and size of stents, thus improving the outcomes of the procedure. The development of artificial intelligence and machine learning with advanced algorithms of prediction will offer multiple scenarios for treatment, allowing real-time selection of the best strategy for revascularization.

## 1. Introduction

Coronary artery disease still represents an important public health issue, despite the advances in the pathology and the treatment of the disease [1]. Coronary angiography remains the gold standard investigation that allows the identification of lesions and severity assessment. Notwithstanding, X-ray angiography is a luminography that simplifies the three-dimensional structure of the vessel into a two-dimensional representation, inducing misinterpretations due to foreshortenings, overlaps or imprecise stenosis assessment in eccentric stenoses. Visual estimation of the coronary percentage diameter stenosis is advocated, together with quantitative comparative analysis, in a recently published complex algorithm for defining the completeness of coronary revascularization [2], but the ability of visual estimation even in experienced operators of the functional significance of a stenosis is reduced with a global discordance of 30% [3], the worst performance being obtained in intermediate lesions [4]. A large burden of the coronary stenoses observed in the catheterization laboratory, or at least the most troublesome, is those classified as intermediate (25% according to a registry [5]), defined by the European Society of Cardiology as being between 50 and 90% diameter stenosis [6], or between 40 and 70% in the American guidelines [7] per visual estimation. This category of lesions raises questions regarding the need for revascularization, inducing in some circumstances biases, mainly because even experienced interventionists can over- or under-estimate an intermediate lesion compared with the measured fractional flow reserve (FFR) [3]. The intermediate coronary lesions inducing ischemia that are left untreated have a higher chance of requiring revascularization in the future, and are associated with poor prognosis compared with non-significant stenoses [8]. This review will discuss the methods used in the catheterization laboratory to guide treatment either towards revascularization or medical approach, and follow-up in intermediate coronary lesions. 

## 2. Fractional Flow Reserve and Instantaneous Wave-Free Ratio

Due to the inconveniences derived from the visual estimation of percentage diameter stenosis, both American and European guidelines recommend functional evaluation either in the form of FFR or instantaneous wave-free ratio (iFR) for intermediate lesions in stable coronary artery disease. Some authors suggest even larger intervals for functional approach than those defined in the mentioned guidelines in the 3V FFR FRIENDS study, as 8.7% of lesions with diameter stenosis have less than 50% presented ischemic FFR value [9]. There are other indications of FFR/iFR apart of intermediate lesions’ functional assessment, specifically the identification of significant (culprit) lesions in multivessel coronary artery disease or in multiple consecutive stenosis in the same vessel due to its high spatial resolution, and measurement of the functional significance of a stenosis in the presence of a distal collateral flow. The estimation of FFR/iFR can also be achieved non-invasively, derived from computational simulations comparable to those invasively measured [10]. 

Fractional flow rate is calculated as the ratio between the distal coronary pressure in the presence of a stenosis and the proximal coronary pressure in the absence of a stenosis during maximal hyperemia. The importance of inducing maximal hyperemia is crucial, as this minimizes the resistance of the microcirculation and therefore represents a good approximation of the coronary blood flow. Herewith, it is worth remembering that FFR is a pressure measurement, not a direct coronary flow determination, and that ischemia potential of a stenosis is also influenced by the total morphology of the lesion, the energy loss to turbulence, friction and separation caused by stenosis, and that all these are drawbacks of the technique. Apart from FFR, another parameter for the functional evaluation of a stenosis is coronary flow reserve is the flow ratio during maximal hyperemia and blood flow at rest. However, there are many limitations of the technique, including left ventricular contractility, heart rate, blood pressure or unsuitable Doppler waves, and therefore no clear cut-off to base the clinical indications. Another important issue to consider in intermediate coronary lesions is microvascular disfunction, as it can induce severe ischemia that can be invasively evaluated in the form of coronary vasoconstrictor as well as vasodilator abnormalities [11]. A recent study from the ILIAS registry (Inclusive Invasive Physiological Assessment in Angina Syndromes) shows a possible role of coronary flow reserve (CFR) in subjects with intermediate FFR of 0.75 to 0.8, where those with CFR ≤ 2.0 and deferred PCI had lower risk of target failure defined as cardiac death, target vessel revascularization or myocardial infarction, compared with those with normal CFR > 2, proving to be a supplementary criteria to strategy patients [12]. Apart from CFR, the index of microvascular dysfunction (IMR) is a better parameter for the evaluation of microvascular resistance and has become the gold standard for the evaluation of microcirculation dysfunction [13,14]. Current commercial wires allow the measurement of both indices of epicardial disease (FFR, RFR) and microvascular dysfunction (CFR, IMR), which are integrated in the same guidewire assuring a more comprehensive evaluation of the intermediate coronary lesions.

Fractional flow reserve or instantaneous wave-free ratio is considered the gold standard for assessing the ischemic potential of a stenosis in stable ischemic heart disease, predicting the clinical benefit of revascularization, but despite the evidence from different clinical trials showing significant reduction to the primary endpoint (death urgent revascularization and myocardial infarction) [15,16,17], and the major indications from the European [6] and American [7] guidelines, its usage is low. In a study by Parikh et al., it is shown that the percentage of subjects with no stress test and intermediate lesions at angiography is very high (61.3%), while in these cases only 16.5% were investigated with FFR [15]. Interestingly, in the same report, reduced FFR usage in daily practice was not dependent on the absence of documented ischemia or the presence of symptoms as expected, revealing that the main reason for underuse remains operator belief in the futility of the technique and not reimbursement policies or training. 

Invasive physiological evaluation with FFR/iFR guidance is recognized as the optimal method to evaluate the functional significance of an intermediate stenosis with superior detection of myocardial ischemia compared to other non-invasive tests [18]. FFR has multiple advantages, such as the high reproducibility and ease of determination in the catheterization laboratory, a clear cut-off of 0.8 and gray zone between 0.75 and 0.8, independence of multiple factors such as microcirculation status, collateral flow or hemodynamic factors (heart rate or blood pressure), and the possibility to subsequently assess the ischemic contribution of multiple stenosis. Besides its hemodynamic significance, FFR can be used to classify coronary atherosclerosis into focal or diffuse, with the use of intracoronary pressure pullbacks, enabling the identification of the most important steps in pressure-drop potential candidate zones for angioplasty. Apart of the advantages of this technology, the method is not without its patient- or lesion-related limitations. The FFR value is determined at maximum vasodilatation, using usually adenosine, but there are also other potent coronary vasodilators such as papaverine or sodium nitroprusside. From here, there are some patient-related disadvantages of the technique, referring to the incapacity of inducing maximal hyperemia, such as in subjects with severe microvascular impairment, or contra-indications to adenosine administration. To overcome the disadvantages of adenosine administration, iFR was developed, measuring the resting pressure gradient at a moment of the diastole called wave-free when the microvascular resistance is low, and therefore meeting the criteria of the FFR. The ADVANCE trial showed that an iFR value of less than 0.89 correlates with to a FFR value of less than 0.8 and proposed a hybrid iFR-FFR approach that reduced the time consumption with functional evaluation [19]. Another drawback of FFR may be the pressure damping induced by the position of the pressure transducer too distally into the vessel, creating normal or false positive results. Furthermore, lesion-specific characteristics may also influence the FFR or iFR calculation, such as ostial or left main coronary artery (LMCA) stenosis or diffuse coronary disease affecting subsequent segments, as well as the clinical situation, acute versus chronic coronary syndromes. Fractional flow rate in acute coronary syndromes shows normal or supra-normal value due to the obstruction of microcirculation with emboli, which is why this is not recommended in acute coronary events, representing one main disadvantage of the technique. For a better evaluation of these cases, intravascular ultrasound (IVUS) or optical coherence tomography (OCT) may be a better solution of complementary solutions. Although FFR is not implemented as a technique to optimize percutaneous coronary intervention (PCI), there are studies showing that sub-optimal FFR post-stenting is associated with future increased vessel revascularizations [20]. Furthermore, FFR remains the best method to appreciate the significance of a jailed side branch after provisional stenting [21].

## 3. Intravascular Ultrasound

Intravascular ultrasound has been used for many years in the assessment of intermediate coronary lesions. This technique uses a piezoelectric transducer that produces ultrasound signals allowing the assessment of the vessel wall and characterizing the atherosclerotic lesions, eliminating the disadvantages of angiography and FFR/iFR. Its main indications in the American College of Cardiology guideline as class IIa recommendation are the assessment of angiographically intermediate stenosis of the LMCA [22] or the mechanisms of stent failure (thrombosis or restenosis) as well as guidance of coronary stent implantation, especially in LMCA or complex coronary artery disease. The European guidelines on myocardial revascularization have similar indications, recommending IVUS for approaching the severity of unprotected LMCA, the mechanisms leading to stent restenosis and guiding PCI. As observed, there is no indication to use IVUS to assess intermediate lesions in non-LMCA, mainly because studies showed only moderate correlation between IVUS-derived parameters and FFR values; for non-LMCA intermediate stenosis physiological assessment remains the gold standard.

Minimal lumen area (MLA) is the best parameter calculated using IVUS correlating with a major adverse cardiovascular event (MACE) [23]. If MLA has a good negative predictive value (75–96%), it has low positive predictive value (28–67%), indicating that it is a good parameter to defer intervention, but should not be used as the only indicator for the indication of revascularization, as this induces unnecessary procedures for lesions without significant functional impact. Although many studies have aimed to find an IVUS cut-off equivalent to the ischemic FFR of 0.8 or 0.75, the MLA thresholds largely differ between studies both in LMCA or non-LMCA. Despite the implementation of different IVUS-MLA cut-offs and refinements according to the type of vessel, location and size [24], the accuracy of detecting an ischemic FFR did not surpass 70%. The accuracy of MLA in the evaluation of LMCA stenosis is higher, most probably due to the simple morphology and dimension of this vessel with no branch involvement in the pure disease of LMCA, its functional significance decreasing when there is complex involvement of the ostial left anterior descending or circumflex artery. For LMCA, the MLA largely varied from 4.5 to 5.9 mm^2^, with guidelines suggesting 6–7.5 mm^2^ threshold for Western subjects according to some prospective multicentric studies showing that a value of 6 mm^2^ or greater is safe to defer revascularization in Western patients [25,26] and 4.5–4.8 mm^2^ more appropriate in Asian patients [27] (Table 1). Although only three studies have researched the optimal MLA-LMCA corresponding to FFR ≤ 0.80 (0.75), the much larger cut-off of 7.5 mm^2^ from the guidelines comes from a registry analysis of patients with normal/minimally affected coronary arteries [26].

Another aspect considered in studies is the inter-ethnic variation in coronary anatomy and size, and thus, a wide range of MLA-IVUS cut-offs between Western and Asian populations, most probably due to lower body mass index and the subtended myocardium for each coronary stenosis. Asian population studies have found an IVUS-MLA for non-LMCA cut-off varying from of 2 mm^2^ in the IDEAS study to 2.97 mm^2^ [38], compared with Western populations, where larger areas were found, from 3.09 mm^2^ [24] to 4 mm^2^ [39]. In a meta-analysis [40] including 14 prospective studies with the aim of evaluating the diagnostic performance of IVUS-MLA to predict significant non-LMCA stenosis, the results showed a mean MLA of 2.68 mm^2^ in Asian populations and 3.03 mm^2^ in Western ones. The same meta-analysis proves that an FFR cut-off of 0.75 is associated with an accuracy four times higher than an FFR of 0.8, used in the majority of studies, but the reason for taking 0.8 as cut-off derives from the desiderate of treating a larger number of lesions without leaving a category of subjects with ischemia uncovered. Even if its accuracy is increased [40], the clinical significance is reduced. 

Another important contribution of IVUS appears when there is important downstream disease of the coronary vessel, since in these clinical situations, the accuracy of FFR is diminished, and a hybrid approach with IVUS and FFR provides essential information not only about severity, but also about vessel architecture in the case of PCI [41].

Apart from MLA, there are other basic parameters such as maximal luminal area, diameter stenosis, plaque burden and lesion length that contribute to the increasing accuracy of IVUS in predicting functional significance [38]. 

Intravascular ultrasound, apart from its variable accuracy in detecting the functional significance of a coronary stenosis depending on numerous factors, has a proven benefit in the guidance of percutaneous interventions, as it allows stent size selection, identification of good proximal and distal landing zones, and post-implantation optimization. Numerous published meta-analyses have shown that IVUS versus angiographic-guided intervention is associated with an overall reduction of MACEs [42,43,44]. Another advantage of IVUS is the possibility for use in acute myocardial infarction compared to FFR, to predict early stent thrombosis or in-stent restenosis as it detects stent under-expansion or a reduced lumen area due to thrombus protrusion, significant edge plaque burden, stenosis or dissection [45]. IVUS-guided PCI is also cost-effective, as shown in an Australian healthcare system study not only reducing costs but also increasing life expectancy and quality-adjusted life years [46]. 

## 4. Optical Coherence Tomography

Optical coherence tomography is the light modality analogue of IVUS, a technique using near-infrared spectroscopy to detect the structure of the vessel wall. Optical coherence tomography brings several benefits over IVUS such as a quicker acquisition of images with higher resolution, characteristics that allow a better identification of dissection, thrombus, plaque ulcerations, stent malapposition and under-expansion. On the other hand, the reduced wavelength of infrared light and the obligatory flush of the catheter to create a blood-free lumen are associated with decreased depth of imaging, mainly in large vessels such as the LMCA, and the impossibility to assess aorto-ostial stenosis, ectacic or tortuous arteries. The procedural time is increased compared with the setting of the system, but automated workflows are imaged to reduce the total times with automated stent identification, lumen or external elastic membrane contour delimitation. The superiority of OCT over FFR in appreciating the severity of coronary lesions is not proven, and therefore not recommended [35,47]. The OCT-MLA cut-offs are smaller compared with IVUS-MLA, and correspond to the true lumen dimensions [48], being superior as ischemia predictors in non-LMCA in some trials [49,50] (Table 2). On the other side of the coin, the smaller measured diameters in OCT may lead to smaller stent diameters and worse long-term outcomes. To overcome this limitation of OCT, several strategies have been proposed such as measurement based on the external elastic membrane rounded to the nearest 0.25 [51] or adding 10% or 0.25 mm to the mean lumen diameter [52]. The OCT guided percutaneous interventions showed decreased edge dissection, malapposition and under-expansion with increased final MLA compared with coronarography, resulting in better post-procedural FFR values in the survey DOCTORS [53]. The high axial resolution of OCT allows the measurement of the thin fibrous cap associated in histopathological studies with increased risk of erosion and rupture. In the COMBINE study, which made use of OCT and FFR, a thin cap fibroatheroma of 60 μm or less was associated with more MACEs in diabetic subjects with intermediate coronary lesions, pinpointing the ability of OCT to detect plaque vulnerability. One trial still recruiting patients is researching vulnerable plaque markers in OCT after acute coronary syndromes, collecting in a prospective manner data about OCT characteristics of plaque vulnerability and their correlation with outcomes [54]. The identification of vulnerable plaques and guidance of medical or interventional treatment intensification upon these might be very useful in the future, although there are no randomized trials showing the improvement of cardiovascular adverse events. 

The comparation between OCT and IVUS in PCI guiding shows contradictory evidence, with some small studies showing that the increased OCT sensitivity in detecting malapposition, for example, does not translate into less adverse events, such as reduced intrastent restenosis or stent thrombosis [63], while others describe a decrease in mortality [64]. The differences in the results of various studies derive from their design, prospective or retrospective, as well as the power to predict clinical outcomes. Although most causes that drive stent failure may be detected by IVUS, neoatherosclerosis, a very important cause of late or very late stent thrombosis, is not detected by it, making OCT the most appropriate technique when this is the presumed mechanism [65]. 

## 5. Emerging Artificial Intelligence Techniques 

The emergent new artificial intelligence algorithms for machine learning might overcome the poor accuracy of IVUS or OCT-derived morphological criteria in predicting ischemia-inducing lesions showing good performance without the expenditure of pressure wires [55]. Mathematical fluid dynamics allows the calculation of several flow reserves, including an OCT-based FFR that has excellent accuracy (95.2%) in predicting the need for revascularization in intermediate coronary stenosis [66], or IVUS-based [55]. Another parameter derived by mathematical assumptions of FFR is virtual flow reserve, with its accuracy being investigated in the FUSION trial (NCT04356027), whose results are still awaited. One limitation to these parameters is that collateral flow is not considered, and might have significant impact in the calculation formula. One non-invasive method to detect ischemia is FFR computer tomography angiography (CTA) using novel computational fluid dynamics. Its good accuracy in predicting lesion-specific ischemia has been evaluated in many trials, such as NXT [67] or DISCOVER-FLOW [68]. Quantitative flow ratio (cQFR) is a parameter obtained from two different angiographic projects that generate a three-dimensional quantitative coronary angiography. Using the Gould formula, the pressure drop is calculated in each segment based on geometry and hyperemic flow velocity. In the FAVOR study, three models were used to compute hyperemic flow, and all of these showed good agreement with FFR [69]. Currently, the cQFR based on the TIMI count analysis without pharmacologically induced hyperemia is the standard displaying better accuracies. Although there is much evidence supporting FFR CTA more than CTA alone in the diagnosis of an ischemia-inducing stenosis, its application is limited due to accessibility, technical limitations and costs to intermediate, calcified stenosis or in cases with artefacts. The coupled CTA and FFR CTA proved to be useful even in subjects with intermediate stenosis and Agatston calcium score of above 400 [67].

New convolutional neural networks have been explored to classify plaques, including the vulnerable ones in OCT [70], as well as in IVUS [71]. Plaque vulnerability predictors, such as the morphological plaque vulnerability index derived from IVUS with machine-learning algorithms, showed good prediction accuracy [72], opening new horizons in the description of morphological features increasing the risk of plaque rupture. A machine-learning model could predict changes in fibrous cap-thickening measured at OCT analyzing the genetic pool of the subject advancing a precise medicine concept [73], while others accurately quantified the cap thickness [74]. Plaque geometry and morphology also play a key role in plaque rupture. Machine learning and three-dimensional reconstruction of plaques can improve the efficiency of some methods, such as CTA in the evaluation of intermediate lesions [75]. Artificial intelligence has an increased potential in making an accurate diagnosis, suggesting personalized treatment based on stratified risks compared to humans, but this domain is still in its infancy, requesting the standardization of methods and quality of data to assure appropriate recommendations.

## 6. Conclusions

One single imaging technique will not answer all the questions raised by coronary angiography in the case of intermediate stenoses. It is worth noting that FFR remains the gold standard for the evaluation of functional significance, but in complex clinical scenarios, it may not prove to be ideal, prompting the interventionist to approach other methods, such as intravascular ultrasound and optical coherence tomography. Each of these has its own inconveniences, but together offer a more comprehensive image of the investigated coronary lesion.

## Figures and Tables

**Table 1:** Intravascular ultrasound-derived optimal minimal luminal area values to predict fractional flow rate in left main coronary artery (LMCA) and non-LMCA.

Study	Vessel	Ethnicity	Lesions	FFR	Best MLA (mm^2^)
Park et al., 2014 [27]	Isolated ostial and shaft LMCA	Asian	112	≤0.80	4.5
Kang et al., 2011 [28]	LMCA	Asian	55	≤0.80	4.8
Jasti et al., 2004 [29]	LMCA	Western	55	≤0.75	5.9
Han et al., 2014 [30]	Non-LMCA	Western/Asian	881	≤0.80	2.75 (Asian) 3 (Western)
Yang et al., 2014 [31]	Non-LMCA	Asian	206	≤0.80	4
Naganuma et al., 2014 [32]	Non-LMCA	Western	132	≤0.80	2.70 (VD < 3 mm) 2.59 (VD ≥3.0 mm)
Cui et al., 2013 [33]	Non-LMCA	Asian	165	≤0.80	3.15
Waksman et al., 2013 [34]	Non-LMCA	Western	367	≤0.80	2.4 (DV < 3.0 mm) 2.7 (VD 3.0–3.5 mm) 3.6 (VD > 3.5 mm)
Gonzalo et al., 2012 [35]	Non-LMCA	Western	61	≤0.80	2.36
Ben-Dor et al., 2012 [24]	Non-LMCA	Western	205	≤0.80	<2.4 (VD 2.5–3 mm) <2.7 (VD 3–3.5 mm) <3.6 (VD > 3.5 mm)
Kang et al., 2012 [36]	Non-LMCA	Asian	784	≤0.80	2.4
Koo et al., 2011 [37]	Non-LMCA	Asian	267	≤0.80	3.0 (proximal LAD) 2.75 (mid-LAD)
Lee et al., 2010 [38]	Non-LMCA	Asian	94	≤0.75	2

FFR fractional flow rate; LMCA left main coronary artery; MLA minimal luminal area; VD vessel diameter.

**Table 2:** Optical coherence tomography-derived optimal minimal luminal areas to predict ischemic fractional flow rate.

Study	Lesions	FFR	Best MLA (mm^2^)	Ethnicity
Lee et al., 2020 [55]	365	0.8	2.3	Asian
Rivero et al., 2020 [56]	41	0.8	1.92	Western
Burzotta et al., 2018 [57]	40	0.8	2.5	Western
Usui et al., 2018 [50]	203	0.75	1.39	Asian
Reith et al., 2015 [58]	142	0.8	1.59	Western
Zafar et al., 2014 [59]	41	0.8	1.62	Western
Pawlowski et al., 2013 [60]	71	0.8	2.05	Western
Pyxaras et al., 2013 [61]	55	0.8	2.88	Western
Gonzalo et al., 2012 [35]	61	0.8	1.95	Western
Shiono et al., 2012 [62]	62	0.75	1.91	Asian

FFR fractional flow rate; MLA minimal luminal area.

## Data Availability

Not applicable.
